# Eye Control Deficits Coupled to Hand Control Deficits: Eye–Hand Incoordination in Chronic Cerebral Injury

**DOI:** 10.3389/fneur.2017.00330

**Published:** 2017-07-17

**Authors:** John-Ross Rizzo, James K. Fung, Maryam Hosseini, Azadeh Shafieesabet, Edmond Ahdoot, Rosa M. Pasculli, Janet C. Rucker, Preeti Raghavan, Michael S. Landy, Todd E. Hudson

**Affiliations:** ^1^Department of Rehabilitation Medicine, New York University Langone Medical Center, New York, NY, United States; ^2^Department of Neurology, New York University Langone Medical Center, New York, NY, United States; ^3^Department of Ophthalmology, New York University Langone Medical Center, New York, NY, United States; ^4^Department of Psychology & Center for Neural Science, New York University, New York, NY, United States

**Keywords:** brain injuries, eye, hand, eye movements, stroke

## Abstract

It is widely accepted that cerebral pathology can impair ocular motor and manual motor control. This is true in indolent and chronic processes, such as neurodegeneration and in acute processes such as stroke or those secondary to neurotrauma. More recently, it has been suggested that disruptions in these control systems are useful markers for prognostication and longitudinal monitoring. The utility of examining the relationship or the coupling between these systems has yet to be determined. We measured eye and hand-movement control in chronic, middle cerebral artery stroke, relative to healthy controls, in saccade-to-reach paradigms to assess eye–hand coordination. Primary saccades were initiated significantly earlier by stroke participants relative to control participants. However, despite these extremely early initial saccades to the target, reaches were nevertheless initiated at approximately the same time as those of control participants. Control participants minimized the time period between primary saccade onset and reach initiation, demonstrating temporal coupling between eye and hand. In about 90% of all trials, control participants produced no secondary, or corrective, saccades, instead maintaining fixation in the terminal position of the primary saccade until the end of the reach. In contrast, participants with stroke increased the time period between primary saccade onset and reach initiation. During this temporal decoupling, multiple saccades were produced in about 50% of the trials with stroke participants making between one and five additional saccades. Reaches made by participants with stroke were both longer in duration and less accurate. In addition to these increases in spatial reach errors, there were significant increases in saccade endpoint errors. Overall, the magnitude of the endpoint errors for reaches and saccades were correlated across participants. These findings suggest that in individuals with otherwise intact visual function, the spatial and temporal relationships between the eye and hand are disrupted poststroke, and may need to be specifically targeted during neurorehabilitation. Eye–hand coupling may be a useful biomarker in individuals with cerebral pathology in the setting of neurovascular, neurotraumatic, and neurodegenerative pathology.

## Introduction

It is widely accepted that cerebral pathology can impair ocular motor and manual motor control. This is true in indolent and chronic processes such as neurodegeneration and in acute processes such as stroke or those secondary to neurotrauma (1–5). More recently, it has been suggested that disruptions in these control systems are useful markers for prognostication and longitudinal monitoring (6–8). Therapeutically, neurorehabilitation strives to address these motor control deficits with approaches that restore ability at the movement level in early intervention and at the functional performance level in later intervention; however, in many cases, movement-level gains do not progress into functional performance-level improvements (9). Cerebral injuries, such as stroke, not only lead to motoric impairments but also sensory limitations; these sensorimotor deficits may compromise visual perception secondary to decreased visuomotor function and lead to difficulties with visually guided action in both the more-affected (contralateral) and less-affected (ipsilateral) hands (4, 10–16). During such experiments manual motor control is often studied objectively and typically without simultaneous eye-movement analysis. However, altered ocular motor function is a sensitive biomarker of brain injury (17, 18) in both the cognitive and motor domains (8, 19) and provides clinical insight into neurovascular, neurotraumatic, and neurodegenerative pathology.

Vision provides primary sensory information during visually guided action. Ocular motor programming controls gaze, which in turn supports the planning of hand movements. Fixations target key spatial positions and are contingent on the functional requirements of the task at hand, such as index finger placement for object manipulation or prehension (20, 21). Additionally, vision-based feedback of the hand is critical to error correction for online control, as gaze updates goal localization and spatial understanding (22). In fact, dependencies between eye and hand have been demonstrated and emphasize the concept of shared planning resources (23, 24). In acquired brain injury (ABI), motor deficits in the limb (e.g., hemiparesis) may be compounded by impairments in ocular motor control (25–33). While manual motor deficiencies are normally evident during clinical examination, ocular motor deficiencies may necessitate objective recording for detection and precise characterization (34–43). If eye and hand movements are quantified simultaneously, an improved understanding of the sensorimotor coupling between vision and eye–hand movement is achievable, and would likely be critical in providing a complete picture of the underlying neurological injury.

The complexity of the coordination between the ocular and manual motor systems is highlighted by the large cerebral network coordinating ocular and manual motor control. The neuroanatomy of human eye-movement control depends on a large interconnected system of cortical and subcortical structures, and includes the frontal eye field, the parietal eye field, the dorsolateral prefrontal cortex, the supplementary eye field, the cingulate eye field, and the basal ganglia (30, 44–58). The neuroanatomy of human reach control depends on the primary motor cortex and the premotor and supplementary cortices, relaying neural information corticofugally through the descending corticospinal tracts to orchestrate hand movements (59, 60). The somatosensory cortex, posterior parietal cortex, cerebellum, and basal ganglia further supplement reach control. The posterior parietal cortex translates visual input and information from the somatosensory cortex into motor programs (60, 61). The extensiveness of these connected networks increases the potential sensitivity of these biomarkers to cerebral damage and highlights the utility of objectifying eye–hand coordination in the setting of neurovascular, neurotraumatic, and neurodegenerative pathology.

Eye–hand coordination centers on the ability to visually encode details in the environment and direct goal-oriented hand movements, including pointing, reaching, grasping, tool use, and object manipulation, encompassing performance in many motor activities relevant to functional independence (62, 63). Precise ocular motor control, resulting in high acuity visual perception, facilitates sound manual motor control, making use of movement-relevant visual inputs (64, 65). Multimodal sensory feedback and sensory predictions in feedforward motor control are essential to visuomotor integration during task-specific movements (66). In neurological injuries, whether neurovascular, neurotraumic, or neurodegenerative, these coordinated motor programs are susceptible to a breakdown or a decoupling between effectors, as a byproduct of specific ocular motor deficits, manual motor deficits, or deficits in the temporal and spatial relationships needed for rapid and integrated motor control. In this study, we tested eye and hand-movement control in chronic, middle cerebral artery (MCA) stroke, relative to healthy controls, in both a visually guided and memory-guided saccade-to-reach paradigm to assess eye–hand coordination. To the investigators’ knowledge, in the setting of ABI, this is the first investigation of objective ocular motor and somatic motor control using an unrestricted, three-dimensional (3D) eye–hand coordination task (67). We hypothesized that chronic hemispheric stroke participants without clinically diagnosed visual deficits on bedside testing would show abnormalities in saccadic and manual motor control, as compared to healthy controls.

## Materials and Methods

### Participants

Thirty participants participated in the research study. There were 17 participants in the control cohort (aged 26.2 ± 4.6), and 13 participants in the stroke cohort (aged 57.4 ± 14.2). Five stroke participants had right hemispheric MCA strokes and eight had left hemispheric MCA strokes. All participants were tested for hand dominance based on the Edinburgh Handedness Inventory (68), and were right-handed. All control participants were right-handed. Two participants were unable to complete the entire protocol and were excluded from the analyses. The clinical characteristics of the stroke participants are presented in Table 1. All participants signed a consent form approved by the Institutional Review Board of New York University’s School of Medicine. The informed consent was created and obtained as per the Declaration of Helsinki (69–71).

**Table 1:** Clinical characteristics of stroke participants.

ID	Age (years)	Sex	H/H^a^	Stroke characteristics^b^	Chronicity (years)	Fugl-Meyer Score^c^
1	78	M	R/L	R middle cerebral artery (MCA) distribution	2.0	66
2	61	F	R/L	R MCA distribution	7.0	66
3	34	M	R/R	L MCA distribution	1.7	66
4	39	F	R/R	L MCA distribution	1.4	45
5	70	M	R/R	L MCA distribution	2.8	58
6	60	F	R/L	R MCA distribution	2.6	30
7	73	M	R/L	R MCA distribution	6.0	58
8	51	F	R/L	R MCA distribution	12.2	30
9	60	M	R/R	L MCA distribution	4.4	63
10	39	M	R/L	R MCA distribution	4.7	47
11	70	M	R/L	R MCA distribution	2.0	66
12	47	F	R/R	L MCA distribution	1.5	61
13	65	F	R/R	L MCA distribution	0.7	66
Average (SD)	57.5 (14.3)				3.8 (3.2)	55.5 (13.3)

*^a^H/H, handedness/hemiparesis: handedness (as assessed by Edinburgh)/hemiparesis laterality*.

*^b^Stroke characteristics, lesion location obtained from medical history with participant and/or family members serving as historian; region and laterality cross-validated for consistency with examination findings*.

*^c^Fugl-Meyer Score, a summation of the Upper Extremity Score (out of 66), which reflects the extent of poststroke motor impairment*.

#### Inclusion Criteria

Participants with stroke met the following criteria: (1) older than 18 years, (2) brain injury in the MCA distribution at least 4 months prior to enrollment, (3) ability to complete the Fugl-Meyer Scale to define arm motor impairment (72), (4) a full range of eye movements in horizontal and vertical directions, as assessed by the experimenter, (5) ability to perform pointing tasks as assessed by a clinician, (6) willingness to complete all clinical assessments, and (7) an ability to give informed consent and HIPPA certifications.

#### Exclusion Criteria

Participants were excluded for: (1) cognitive dysfunction less than 24 on the Folstein Mini–Mental Status Exam (73), (2) significant injury to the eye, weakness in extraocular muscles or presence of visual field cuts, (3) hemi-spatial neglect, (4) major disability, as determined by a score greater than 4 on the modified Rankin scale (74), (5) previous neurological illness, confounding medical conditions or significant injury to the upper extremity, (6) significant depression determined by a score less than 11 on the Geriatric Depression scale (75), (7) pregnancy, and (8) electrical implant devices, e.g., pacemakers or defibrillators.

A focused stroke history and neurological and musculoskeletal examinations were performed on all participants. Visual impairments were assessed by the Beery-Buktenica Developmental Test of Visual-Motor Integration (Beery VMI) (76–78), by standard clinical tests for visual acuity (Snellen chart) (79) and visual fields (confrontation and if in question, Goldman or Humphrey visual field testing) (80). Participants were also assessed for hemi-spatial neglect *via* the Schenkenberg’s line bisection test (81) and the single-letter cancelation test (82). Lastly, the 25-item National Eye Institute Visual Functioning Questionnaire and a 10-item supplement survey were completed to quantify the extent of disability due to perceived visual deficits (83).

### Apparatus

#### Monitor and Physical Configuration of the Rig

**Figure 1:**
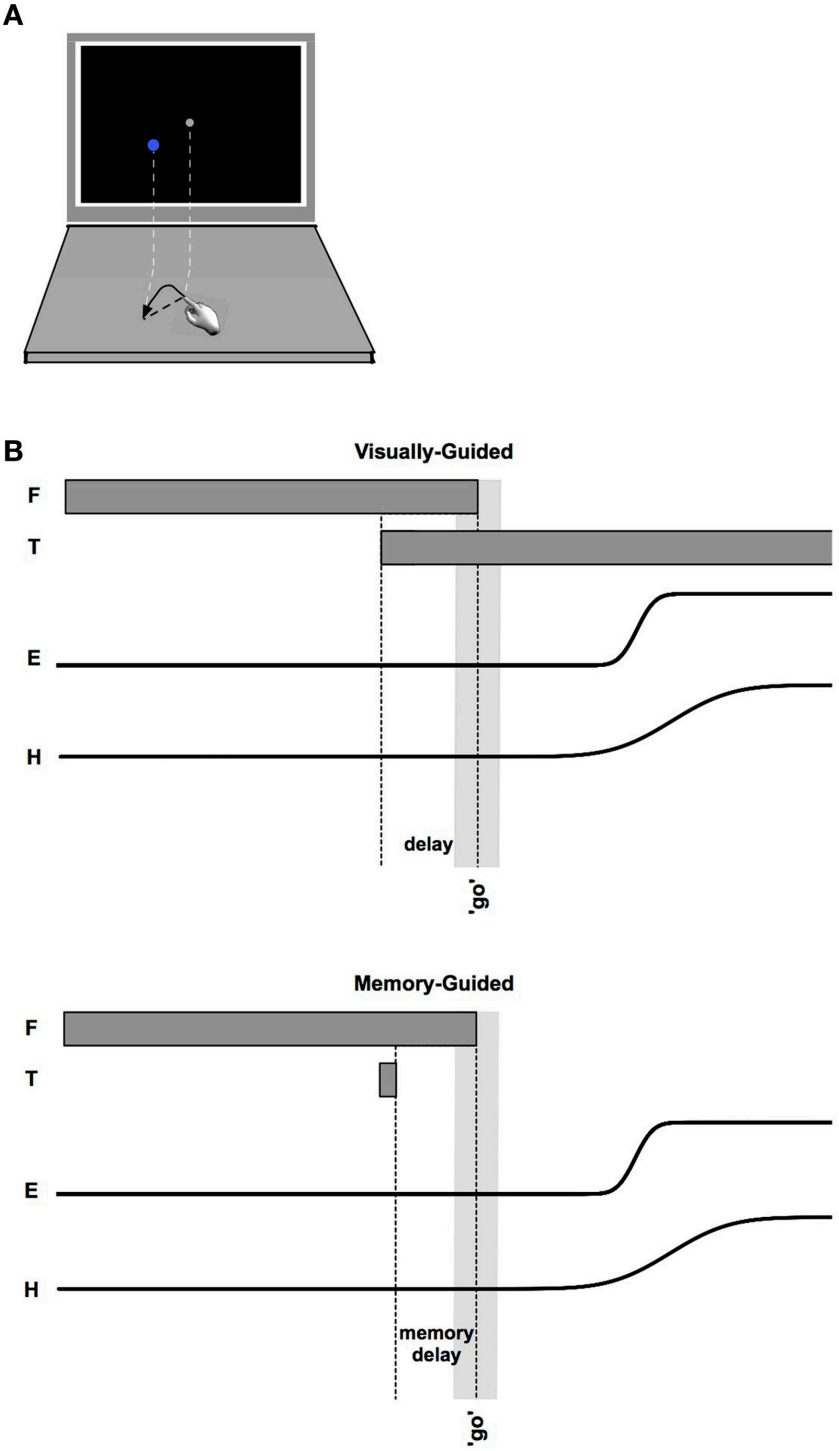
**(A)** Schematic of monitor and tabletop during a reach. **(B)** Sequencing of events within visually guided (upper) and memory-guided (lower) trials. Fixation (F) appears first. After an unpredictable length of time, the target (T) appears. The “go” signal (simultaneous offset of F and an auditory beep) occurs after a variable time interval following target onset (indicated by the light gray vertical bar). Eye (E) and hand (H) movements follow the go signal.

Participants sat at a table with a computer display (19.5″ Dell D2015H LED monitor, resolution 1,920 × 1,080) 60 cm away. A 43.5 cm × 23.5 cm rectangle, identical in size to the computer monitor, was outlined on the table surface between the participant and the display. Participants sat centered to the horizontal length of the screen in a height-adjustable chair. Participants were seated approximately 60 cm from the screen and 40 cm from the table-mounted eye tracker. This physical configuration of the table surface and monitor allowed participants to simultaneously view the screen and make point-to-point reaches on the tabletop (Figure 1A).

#### Computer and Software Program

An ASUS ROG G750JM 17-Inch Gaming Laptop (AsusTek Computer Inc., Taipei, Taiwan) was utilized for this experiment. Custom Matlab (MathWorks, Inc., Natick, MA, USA) scripts, making use of additional functions from the Psychophysics Toolbox (84), were used to display visual stimuli and perform real-time integration of data acquired from the Tobii eyetracker and Polhemus limb tracker.

#### Eye and Limb Trackers

The Tobii X120 eyetracker (Tobii, Danderyd, Sweden) was used to record gaze position (120 Hz, 0.5° accuracy). Kinematics of the finger were measured using a Polhemus Liberty™ 240/16 (Polhemus, Colchester, VT, USA), and Polhemus MicroSensor 1.8 (240 Hz, 0.08 cm accuracy). The motion sensor was affixed to the distal aspect of the index finger of the hand on the to-be-tested arm (the dominant arm for controls, and both arms in participants with stroke). The Polhemus sensor was affixed to the finger by first placing it on the finger and securing it at three locations (proximal and distal phalanx and wrist), using soft flexible neoprene mini-sleeves that were affixed with Velcro and custom fit to each participant.

### Procedure

#### Calibration

The Polhemus output was calibrated to the space occupied by the virtual screen represented on the tabletop using a 9-point calibration. The fingertip location was found relative to the sensor by asking participants to place their fingertip at a known tabletop location (relative to the calibrated “virtual screen” coordinate system).

An authentication procedure verified that the distance from the screen to the participants’ eyes was 60 cm, and an 11-point spatial calibration of the eyetracker was completed (1 center point and 10 equidistant points around a 7.62-cm virtual circle were fixated in random order). The eyetracker calibration was performed twice per session, at the start of the experiment and at its halfway-point.

#### Experiment

After completion of the inclusion/exclusion questionnaires and consent forms, participants were instructed to: “touch a series of tabletop locations as displayed on the computer screen, performing combined look-and-point movements as accurately as possible within the allotted time.” Participants were also instructed to make a “true” pointing movement from the start position to the target (lifting the hand and finger in the process), rather than dragging the fingertip across the surface (as if drawing). Participants initiated the task only after the experimenter confirmed that they understood the task and the 1:1 relationship between the computer screen and tabletop.

Participants performed either center-in or center-out reaches on the tabletop as instructed by the visual display. Start points and targets were chosen from a set of six locations: one at the screen center, and the remaining five located on a circle of diameter 7.6 cm. Starting points (gray) and targets (blue) were displayed as circles of 1 cm radius. The position of the finger was represented on screen as a red dot of 4 mm radius. Finger position was displayed in real-time starting 500 ms after the last reach ended, until the following target was displayed.

At the beginning of each trial, participants moved their finger onto the start position, covering the start circle on the screen with the finger-indicator dot. Maintaining finger position, participants were required to fixate the start position on the screen. If at any time the finger or eye left the start position before the go signal, the screen flashed red (50 ms) and the trial restarted. Once the fingertip indicator and fixation were maintained at the start position for 150 ms, a target appeared. There were two conditions (Figure 1). In the memory-guided condition, the target was flashed for 100 ms. In the visually guided condition, the target was displayed prior to the go signal and remained illuminated until the end of the trial (i.e., a delayed-saccade task) (85) (note that the pattern of results reported below was the same in these two conditions, and were combined). These two saccade-to-reach paradigms were utilized in this experimental setting to increase exploration of the neuroanatomical saccade network during objective testing.

For both paradigms, participants were required to continue fixating the start position (not the blue target) until a “go” beep sounded and the start position disappeared, and then to move both their eyes and fingertip quickly and accurately to the designated target. To prevent anticipation of the go signal, the duration of the delay between presentation of the target and the go signal was unpredictable, ranging from 250 to 750 ms. The end location of the reach was determined by a combined low-velocity (<5% peak) and 3 mm *z*-plane threshold and was displayed as a white dot.

Prior to starting data acquisition, a series of familiarization trials was performed. The familiarization period ended when participants successfully touched 5 of the 10 most recent targets. This performance criterion was meant to insure that all participants understood the procedure and were able to complete the required reaches and eye movements. Following familiarization there were two halves to the experiment (76 look-and-points in each). In one half, reaches all began at the central position and targets were chosen randomly from the five peripheral locations. In the other half, start positions were chosen randomly from the five peripheral locations and the target was always the central position. The order of the two halves of the experiment was randomized across participants.

Whenever possible, participants with stroke performed the experiment with both the more-affected and less-affected arms. Participants who did not feel capable of performing the experiment with the more-affected arm participated with the less-affected arm only. One participant completed the more-affected side session and did not return for the scheduled less-affected side session; three participants completed the less-affected side session and did not return for the scheduled more-affected-side session. Two participants dropped out and two participants were unable to complete the entire protocol for a given session and related data were excluded.

### Statistical Analysis

Raw eye- and hand-position data were initially filtered by a 3-point median filter to remove outliers. Kinematic data traces were then obtained by first aligning data to the time of reach onset. Velocity traces were unremarkable, and are not explored further.

Two-sample *t*-tests were used to determine whether pairs of means or variances differed. Our results were unchanged if comparisons were made using Welch’s *t*-test, which makes use of equations designed to account for possible heteroscedasticity and unequal sample sizes (the Welch-Satterthwaite equation for degrees of freedom). As a complement to traditional *t*-tests, we have plotted Bayesian 95% confidence regions around all computed estimates in the figures; as can be seen graphically in the corresponding figures by comparing confidence bounds, Bayesian analogs of the reported *t*-tests confirm our statistical analyses. Single proportions were compared via the *z*-test for equality of proportions (*S*_1_ of *N*_1_ vs. *S*_2_ of *N*_2_), where *z* is

$$z=\frac{S_{1}/N_{1}-S_{2}/N_{2}}{\sqrt{\left(\frac{S_{1}+S_{2}}{N_{1}+N_{2}}\right)\left(1-\frac{S_{1}+S_{2}}{N_{1}+N_{2}}\right)\left(\frac{1}{N_{1}}+\frac{1}{N_{2}}\right)}}.$$

Finally, we note that separating temporal and spatial errors by target directions either toward or away from the more-affected side (i.e., away or toward the affected hemisphere) did not affect the pattern of results described below.

## Results

### Demographics and Questionnaire Assessments

The clinical characteristics of the participants with stroke are presented in Table 1. The ABI cohort had a mean unweighted VFQ score of 91.33 ± 13.01 vs. 94.87 ± 4.87 in healthy controls (*p* = 0.203, ns). For the 10-item supplement, the ABI cohort had a mean score of 95 ± 11.57 vs. 96.27 ± 6.64 in controls (*p* = 0.375, ns). For the composite and 10-item supplement, the ABI cohort had a mean score of 92.36 ± 12.18 vs. 95.12 ± 4.65 in controls (*p* = 0.244, ns). In the ABI cohort, the mean Fugl-Meyer Score was 55.54 ± 13.33, with a range of 30–66.

### Latencies and Durations of Eye and Hand Movements

**Figure 2:**
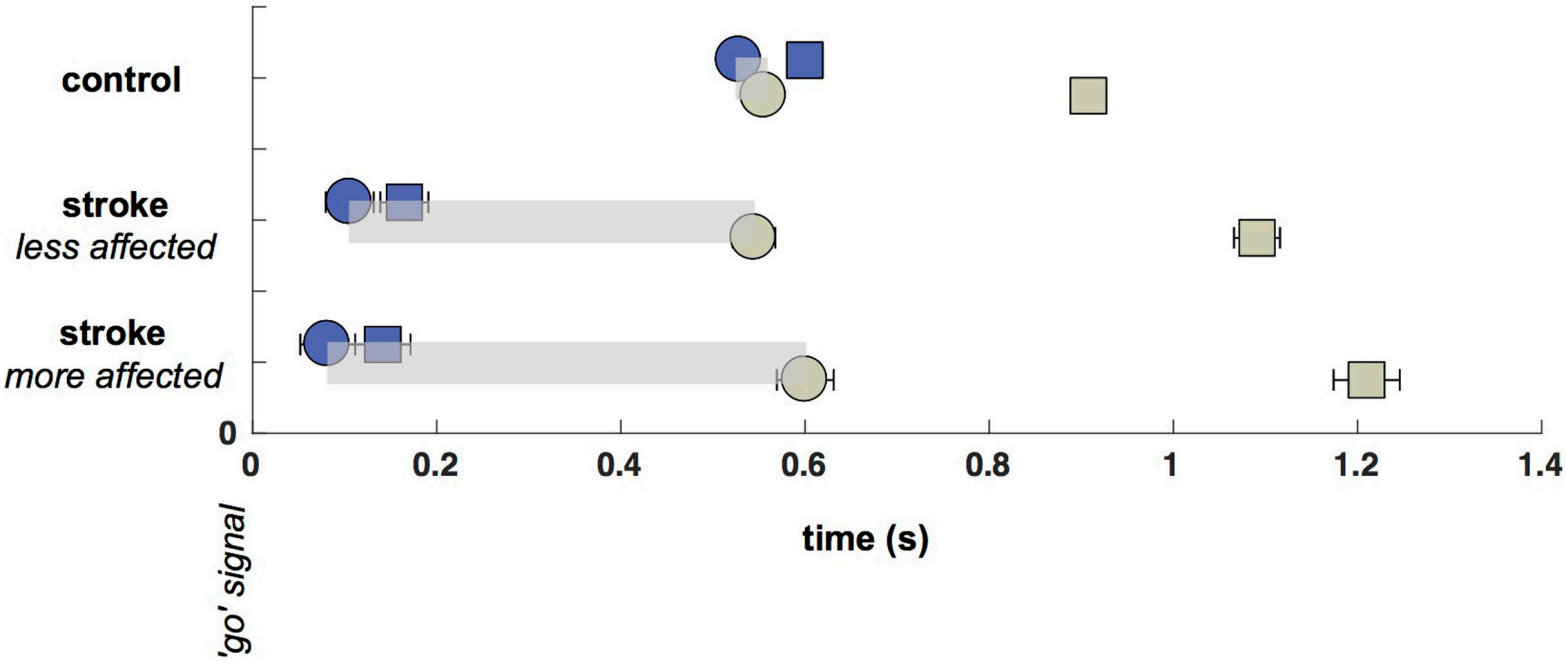
Saccade and Reach Latencies (onsets: circles, terminations: squares). Saccade onsets (blue circles) occur substantially earlier in the stroke cohort, although reach onsets (green circles) are nearly the same across participants regardless of cohort or laterality (with a small delay on the more-affected side). Time between saccade and reach onsets is shown with a light gray bar.

Saccade and reach latencies are plotted in Figure 2 relative to the go signal. Note that the initial (primary) saccades made by participants with stroke are significantly earlier (*p* < 0.05, comparing controls to both less-affected and more-affected sides) than those of control participants [control saccade onsets: 0.529 s, CI: (0.514, 0.543); less-affected arm: 0.106 s, CI: (0.08 0.132); more-affected arm: 0.082 s, CI: (0.052 0.112)]. However, despite the extremely early initial saccades to the target by participants with stroke, reaches were initiated at approximately the same time [no significant differences between control and either less-affected or more-affected reach onsets: control reach onsets: 0.556 s, CI: (0.544 0.568); less-affected arm: 0.545 s, CI: (0.521 0.568); more-affected arm: 0.60 s, CI: (0.567 0.632)].

The temporal decoupling, defined as the interval between the primary saccade and reach onset, is clearly increased in stroke. The coupling between eye and fingertip onsets in controls was 27 ms [CI: (8.5 45)], whereas there was a 439 ms [CI: (404 474)] separation for the less-affected side in stroke, and a 519-ms [CI: (476 562)] separation for the more-affected side in stroke (differences between pairs of coupling times were all significant, all *p* < 0.05). Thus, there was a decrease in coupling with reduction in arm motor capacity or an increase in arm motor impairment (from control to less-affected and less- to more-affected limb reaches in stroke). While it is not surprising that reaches made by the more-affected arm in stroke were prolonged relative to controls [604 ms, CI: (587 622) vs. 352 ms, CI: (348 356)], reaches made with the less-affected arm were also significantly prolonged relative to controls [546 ms, CI: (537 555) vs. 352 ms]. In addition, more-affected-side reaches were prolonged relative to less-affected side reaches (all *p* < 0.05).

### Frequency of Eye Movements

**Figure 3:**
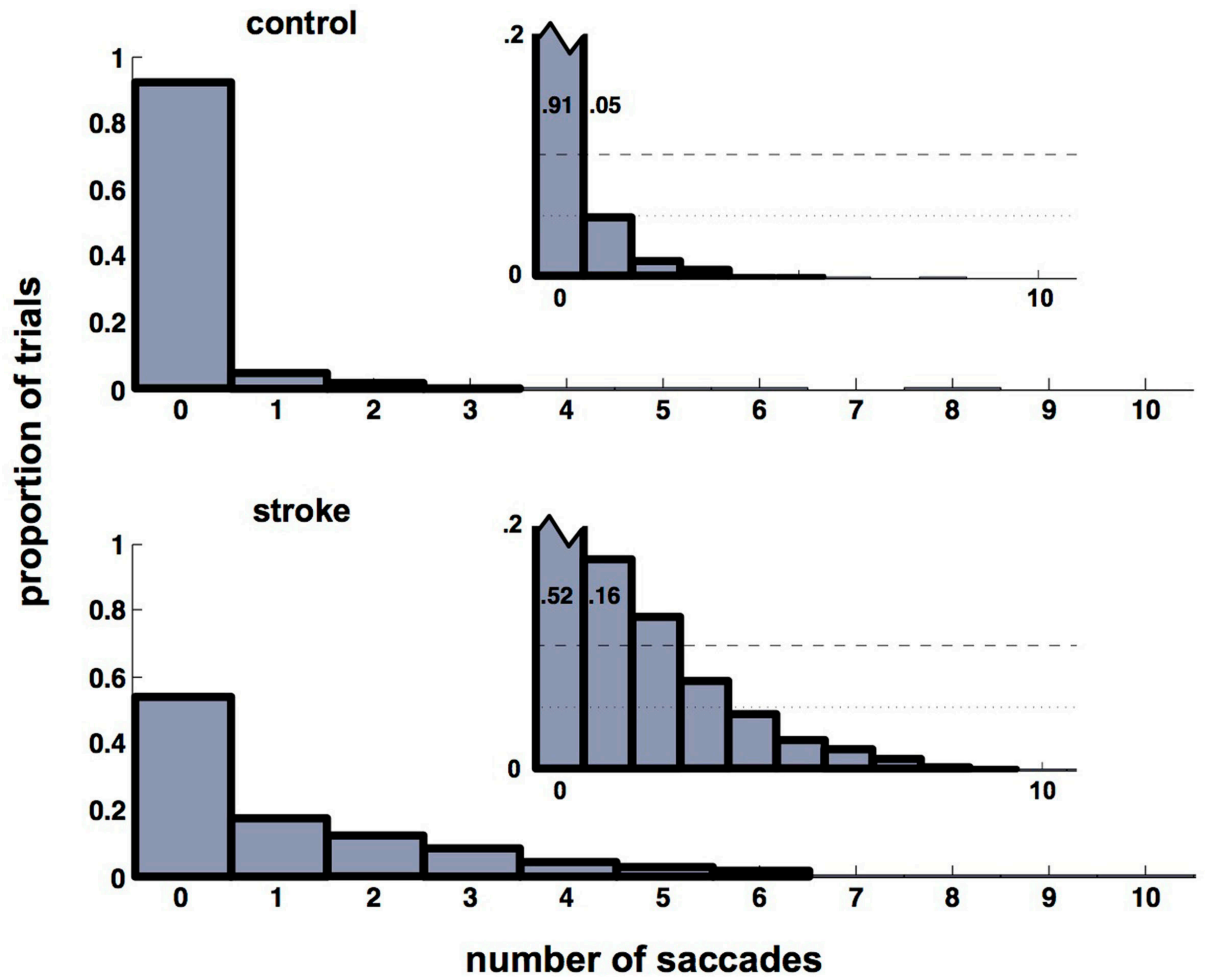
Histograms of the number of saccades in addition to the primary saccade. Control participants (upper histograms) overwhelmingly produce a primary saccade only (91% of trials). About 96% of trials contain either no additional saccades beyond the primary saccade, or contain a single secondary saccade (see inset). For stroke participants (lower histogram), the same 96% of trials contains up to five secondary saccades (see inset). Insets show the same histograms with re-scaled axes to highlight histogram heights for non-primary saccades. This re-scaling truncates the ordinate at *p* = 0.2, which allows the pattern in the smaller-height histogram bars (those corresponding to trials that included non-primary saccades) to be seen. Heights of the first two bars in each inset are labeled to help emphasize the re-scaling.

**Figure 4:**
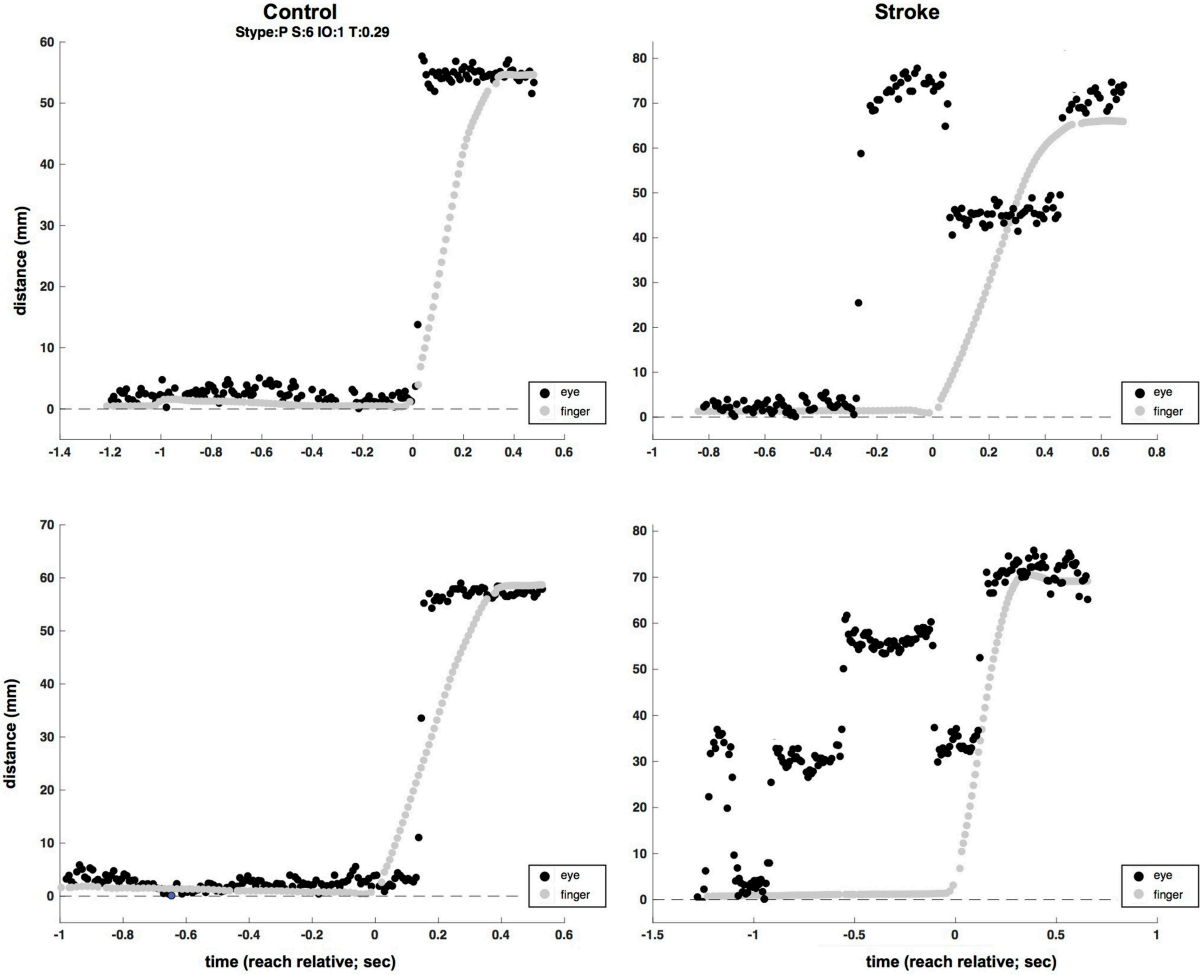
Two-sample (raw, unfiltered) eye (black) and hand (gray) traces from control (left column) and stroke (right column) participants (plotted in screen mm to allow for simultaneous plotting of eye and hand traces). Multiple eye movements are evident in the time before reach completion in the two stroke trials, as opposed to single saccades at or near the time of the reach in control trials.

The significant delay between initial saccade and reach onset in both the more- and less-affected sides of stroke participants relative to a minimal saccade-reach temporal separation in control participants suggests that an important temporal decoupling has occurred. Therefore, we examined the time period between the primary saccade and initiation of reach. Participants with stroke frequently made multiple saccades between the start and target positions (this pattern was the same in more- and less-affected arm reaches, and these are combined here), rather than a single saccade as seen in control trials. Figure 3 displays histograms of the number of additional saccades (past the initial, or primary saccade) that were made by each group. Note that control participants overwhelmingly produced a single (primary) saccade to the target and maintained fixation in the terminal position of the primary saccade until the end of the reach in approximately 90% of all trials. In stark contrast, stroke participants generated this pattern in only about half of trials (*z* = 32.2, *p* < 0.05); these participants commonly produced from one to five additional saccades (Figure 3). Example saccade traces illustrating this phenomenon are shown in Figure 4.

### Spatial Errors of the Eye and Hand Movements

**Figure 5:**
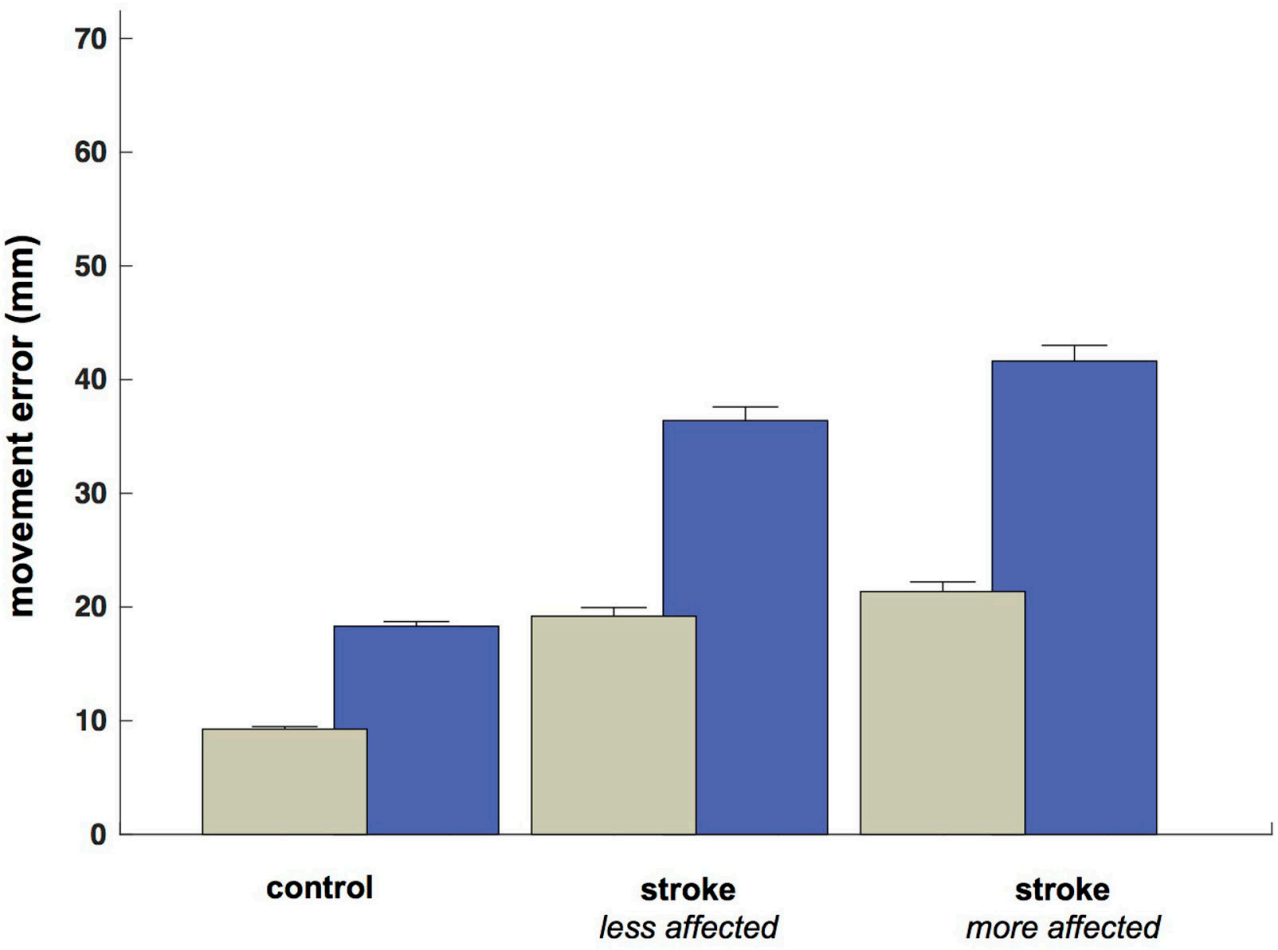
Average endpoint error by participant grouping and/or arm (mm at the screen). Green bars show average reach error, and blue bars show average (primary) saccade error.

**Figure 6:**
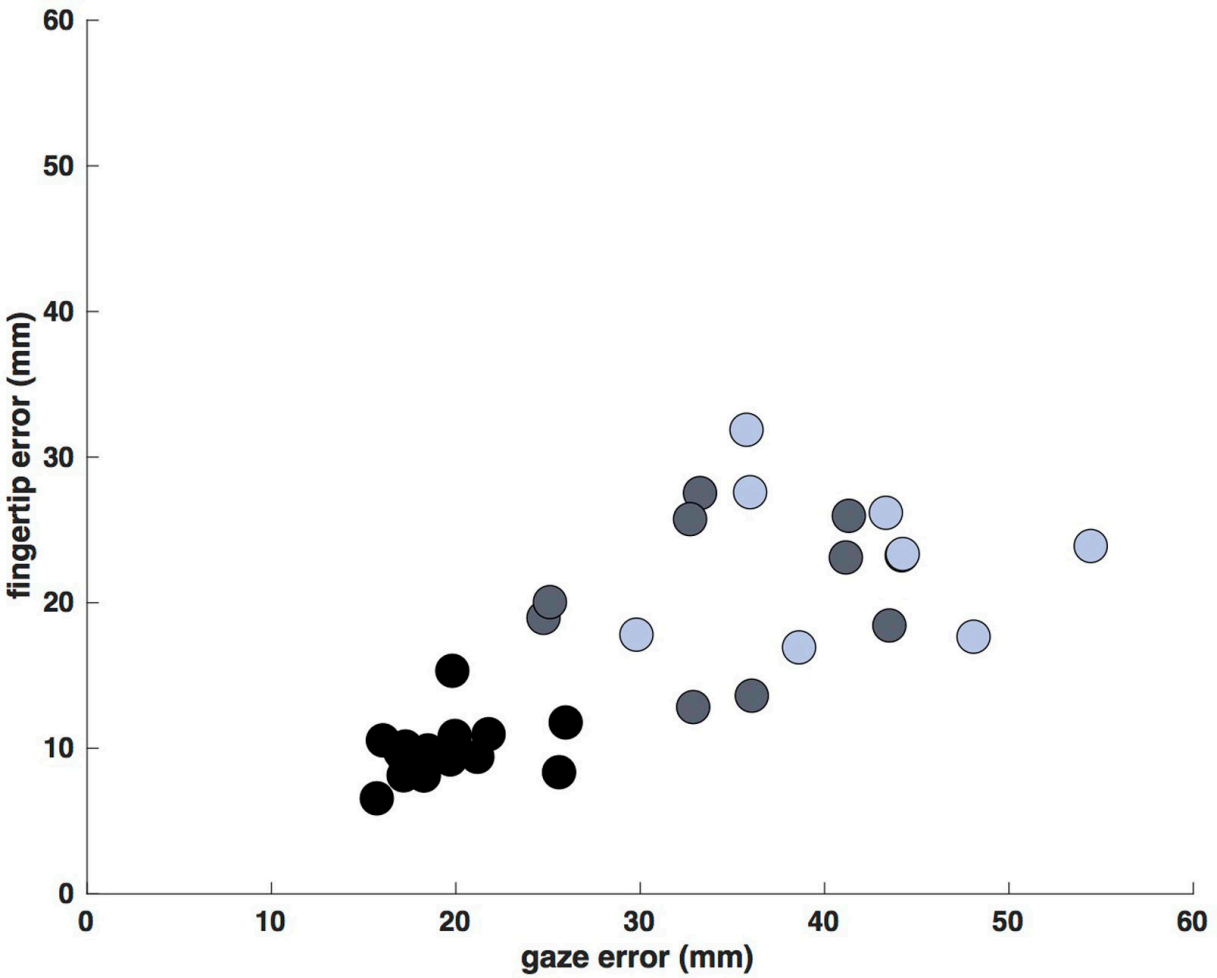
Average saccade vs. reach endpoint error (mm at the screen). Each data point is the average error for a single participant/arm (control movements: black, less-affected arm: gray, more-affected arm: blue). Errors display a dependence on arm motor impairment, generally increasing across participants from control to stroke, and from less- to more-affected limbs (*r* = 0.76, *p* < 0.05).

Despite the increased duration of reaches in the less- and more-affected arm trials relative to control trials (allowing for a greater degree of feedback control), spatial errors (reach endpoint distance from the target) increased in stroke participants [control: 9.3 mm, CI: (9.0 9.5); less-affected arm: 19.2 mm, CI: (18.4 20.0); more-affected arm: 21.4 mm, CI: (20.5 21.4)] rather than decreased (Figure 5; all *p* < 0.05). In addition to these increases in reach error, Figure 5 shows even larger increases in saccade endpoint error [control: 18.3 mm, CI: (17.9 18.7); less-affected arm: 36.4 mm, CI: (35.2 37.6); more-affected arm: 41.6 mm, CI: (40.3 43.0); all *p* < 0.05]. Figure 6 shows the correlation between gaze and reach endpoint errors across subjects. Saccade and reach errors are correlated (*r* = 0.76, *p* < 0.05) across participants and levels of arm motor impairment.

### Correlation between Arm Motor Impairment and Eye–Hand Latency Decoupling

We then asked if the extent of eye–hand decoupling was larger in participants with greater arm motor impairment (lower scores) as assessed by the Fugl-Meyer Score. Although the predicted trend is in fact observed, it is not statistically significant for the less-affected (*r* = −0.64, ns) or more-affected (*r* = −0.34, ns) arms.

## Discussion

We have demonstrated a number of findings in eye–hand coordination after stroke in individuals with otherwise intact visual function. Most important among these results is the temporal decoupling between the primary saccade onset and the reach onset in the saccade-to-reach tasks. Saccades and reaches in stroke participants were also less accurate regardless of reaching limb (more- or less-affected side), as compared to controls. We discuss each of these findings in turn, paying particular attention to the clinical implications these results may have on eye–hand coordination in the setting of neurovascular, neurotraumatic, and neurodegenerative pathology.

### Temporal Decoupling and Latency Abnormalities

The temporal decoupling between eye and hand is clearly noted in the latency differences for both the less-affected and more-affected reaches poststroke. While there are several important elements to extract from the timing data, the most substantial finding was that saccades made by stroke participants occurred significantly earlier in both the less-affected and more-affected arms, as compared to the saccade onsets of control participants (Figure 2). This is consistent with earlier reports of an upper-motor-neuron-like disinhibition phenomenon, in which participants with cerebrovascular damage anticipate the movement go signal, notwithstanding instructions to the contrary (86). Despite extremely early primary saccades to the target, reaches by stroke participants were initiated at roughly the same time as those of control participants, yielding the temporal decoupling that distinguished our cohorts (Figure 2). Thus, temporal decoupling appears to be a result of the unusually early onset of initial saccades, rather than due to the late onset of arm movements.

The eyes frequently fixate an object of interest before starting a manual motor movement (87); though, a more invariant feature is that gaze is spatially directed to the target prior to the arrival of the hand (88), typically close to the peak acceleration of the reach (89–91). The ocular motor system controls the gaze that then provides the needed visual information to optimally direct the hand; this is performed so fixations are “just in time,” providing information at a critical moment, during which additional fovea-based fine detail is required for the task (92). Additionally, the short-term memory limitations of visual features are well known aspects of visual function and further support the idea that information acquired during prior fixations factors marginally into the computations necessary for ongoing fine motor control (93, 94). The information that is used across fixations within a visual scene is principally semantic in nature: for example, the memory of a global environment but not specific details (95, 96). Consequently, eye movements are intimately coupled in time and space to the motor action of the hand (97).

Vision may be best understood through action production, as sensorimotor coupling involves the distillation of visual perception into defined benefits for the planning and execution of somatic behavior (98, 99). As previously detailed, gaze is often directed at environmental objects with relevance to future action; in particular, during object manipulation the line of sight is directed at spatial targets upon which manual interactions may subsequently be focused (20, 100, 101). Complex, manual interactions with an object have multiple stages (e.g., stage 1: reach for the object; stage 2: grasp it; stage 3: lift and maneuver it), such as might occur when one reaches for a bottle of water lying on its side, then lifts it from the table and finally re-orients it for ease of grasp by a colleague. All stages of such a complex task have significance not only for the planning and motor control of the hand position but also for the planning of gaze, suggesting that manual activity “stages” can differentially affect eye position (59). For example, adding weight to the hand during a visually guided reach (in an effort to up-regulate the motor command and efference copy) modulates saccadic output (102).

These examples illustrate a two-way flow of information between eye–hand and hand–eye, which may be particularly relevant in pathology with arm motor impairments. This may be compounded, as demonstrated here, when visual information is not timed correctly and is decoupled from manual motor activity, limiting the opportunity for relevant visual information to support the evolving manual motor planning necessary for accurate reaching. However, to understand the full progression of reaches (and reach errors) generated in the present experiments, one must understand the planning deficits and errors generated. These spatial accuracy compromises may be a byproduct of impaired planning, feedback, and/or online (feedforward) corrective mechanisms.

### Spatial Errors and Predictive Control

Despite increasing reach duration in the less- and more-affected arm trials relative to controls, theoretically allowing additional time for feedback control mechanisms to take effect, spatial errors increased. The fact that there was an increased opportunity for feedback mechanisms to reduce reach errors and yet these errors increased may indicate that feedback mechanisms produced inappropriate trajectory “corrections” and caused increased errors, or inappropriate plans were activated for a given reach while feedback mechanisms were suppressed, or poor estimates of reach errors were generated, or a combination thereof. In addition to increased reach error in stroke, there was an even larger increase in saccade endpoint error from controls to less-affected and then more-affected limbs in stroke.

It is particularly interesting that there was such a large decrement in saccade accuracy between the control and less-/more-affected arm reaches. This increase in endpoint error may have been a byproduct of the increased frequency of saccades, as stroke participants were found to elicit multiple saccades between the start and target position, rather than a single saccade as was found in control trials. In fact, stroke participants commonly produced between one and five additional saccades relative to controls in the time period before reach termination (Figures 3 and 4). This behavior is akin to what might be seen under “normal” conditions when one, given some degree of uncertainty, attempts to visually estimate the length of an object or distance. Although saccadic dysmetria has been documented and ascribed to lesions involving the cortex, pretectum, thalamus, superior colliculus, and cerebellum (103–105), we are unaware of any previous example in the literature of the above ocular–manual behavior occurring under experimental conditions, nor any report of it arising in a participant population with ABI undergoing an investigation of eye–hand coordination.

Prediction is an essential component of goal-oriented somatic action; the physical world is constantly changing and consequently an important aspect of eye–hand control. Grasping a cup being given to you requires both anticipating the object’s direction and motion, and planning a motor response that predicts the trajectory to successfully intersect with it. If visual perception were merely used to generate 3D cues for eye–hand coordination, our fingers would regularly miss their spatial goal due to poor predictions of objects and/or hand motion. Prediction is required for optimized motor control, which translates into functional performance (106). These principles are most clearly highlighted in sports, where athletes of higher skill demonstrate finely tuned ocular motor control with predictive capacity, driving superior, complex, somatic motor control (107–111). For instance, expert-level soccer goalkeepers can more accurately predict soccer ball trajectories during anticipation tasks and leverage more efficient and effective strategies during visual search when compared to novices (112, 113).

As a pathologic illustration, optic ataxia manifests with an inability to efficiently adjust online hand trajectories targeted at moving spatial targets or to properly reach for/grasp objects under visual control. These deficits in rapid error corrections and their mechanistic underpinnings shed light on the coupling required between eye and hand during visually led function (114). In ABI, impairments are prominent during dynamic eye–hand coordination tasks, emphasizing potential difficulties in rapidly processing sensory information, sensorimotor integration and planning, in addition to motor execution. Inefficiently handling sensory information may lead to difficulties in predicting target motion, a deficit in feedforward mechanisms, and in the integration of sensory feedback toward error correction (3, 115). In fact, predictive control is vital to optimized visuomotor planning (116). It is presently accepted that impaired planning is a result of an inability to program motor action sequences in space and time (10, 117–120), and, that post-ABI there are deficits in the motor programming necessary to plan for static or dynamically moving targets (121–124). We believe our findings to be consistent with these prior results and may suggest why these deficits are apparent in both the less- and more-affected sides.

### Clinical Implications and Outcomes

Here, we describe a pattern of abnormalities following MCA stroke that affects both eye–hand coupling and sensory-motor performance, where the strength of the deficit increases for reaches made with the less-affected to reaches made with the more-affected arms of stroke participants relative to the baseline performance in control participants. These findings suggest that in individuals with otherwise intact visual function, the spatial and temporal relationships between the eye and hand are disrupted poststroke, and may need to be specifically targeted during neurorehabilitation. Eye–hand coupling may be a useful biomarker in individuals with cerebral pathology in the setting of neurovascular, neurotraumatic, and neurodegenerative pathology.

Quantitative eye-movement analysis has proven to be a high-value research tool within ABI (49, 125, 126); objective ocular motor recordings have even been used for screening in a diagnostic capacity (127–130). In a broader scope, eye movements and the upper limb have been sensitive markers of cerebral injury when examining visuomotor skill (131). Additionally, function of the eye and arm following acute ABI can predict outcomes in the subacute and chronic stages following injury, with greater performance when compared to self-reported health status or neuropsychologic assessment (3, 132, 133). These prognostic capabilities extend to the identification of individuals who may require more comprehensive intervention or who are poor responders (6, 7). In fact, eye-movement findings have even been shown to be a biomarker of cognitive recovery beyond the times at which presumed full recovery had been reached, as assessed by established metrics (8). While the evidence is greater for neurotraumatic and neurovascular etiologies, the literature base also includes neurodegeneration, in which eye movements may be a biomarker of progression and useful in clinical trials of pharmacological agents to slow disease advancement (134–137). At the bedside, regardless of whether a clinical assessment of visual function is found to be remarkable or unremarkable, as was the case in our pathologic cohort following stroke, disruption of the normal coordination between ocular and manual motor control may lead to maladaptive compensation strategies. This dysfunctional, compensatory behavior, which may require objective screening, and be evidenced by increased saccade frequency during temporal decoupling, may lead to problems in either motor planning and/or control systems and have untoward consequences on function.

It is paramount to remember that sensorimotor control strategies are critical for skilled somatic behavior in humans and that disruptions leading to incoordination may ultimately hamper recovery following ABI. Visuomotor integration is characterized by temporal and spatial relationships between the ocular and manual motor systems (138, 139); small abnormalities in eye-movement timing relative to hand-movement timing, irregularities that could go undetected, may disrupt the framework on which combined movement plans are constructed (140). Moreover, eye-movement execution for visually guided reaches may occur concurrently with motor planning for limb movement (141, 142). This could add in a compound fashion to the already known motor planning deficits in chronic stroke (123), generating computational delays and providing a potential explanation for stifled rehabilitation progress and recovery plateaus. As ocular motor control precedes and is an integral component of visually guided limb control (138, 139, 143, 144), eye–hand coordination is critical to function. Understanding the synchronous and interdependent control systems that direct the eye and hand will likely be important to restoring upper extremity function poststroke. Within neurorehabilitation, one must remember that there is a key difference between gross motor ability and functional motor control. The distinction between these two sides of recovery is not the simple capacity to move the limb but rather the character and efficiency of that control.

## Conclusion

Despite the robust opportunities within ocular–manual motor investigations in the setting of ABI, examination with quantitative dual-effector recordings in 3D has not been formally tested. We report on a number of findings in chronic, MCA stroke, relative to healthy controls, in visually guided (delayed) and memory-guided saccade-to-reach paradigms to assess eye–hand coordination. As compared to healthy controls, stroke participants demonstrated significant temporal decoupling between primary saccade and reach onsets, greater endpoint errors in both effector systems (poorer spatial performance), and an increased frequency of saccades during the temporal decoupling. Future studies that further characterize coupling objectively in unconstrained and naturalistic tasks with ecological validity may produce high-yield results for neurorehabilitation in the setting of neurovascular, neurotraumatic, and neurodegenerative pathology.

## Ethics Statement

This study was reviewed and approved by the Institutional Review Board of New York University’s School of Medicine. Written informed consent was created and obtained as per the Declaration of Helsinki.

## Author Contributions

Conception and design of the study and substantial manuscript drafting: J-RR, JF, MH, AS, EA, RP, AA, PR, JR, ML, and TH. Acquisition and analysis of data: J-RR, JF, EA, MH, RP, AS, and TH.

## Conflict of Interest Statement

The authors declare that the research was conducted in the absence of any commercial or financial relationships that could be construed as a potential conflict of interest.

## References

[1] Tippett WJ Krajewski A Sergio LE. Visuomotor integration is compromised in Alzheimer’s disease patients reaching for remembered targets. Eur Neurol (2007) 58:1–11. 10.1159/000102160 17483579

[2] Verheij S Muilwijk D Pel JJ van der Cammen TJ Mattace-Raso FU van der Steen J. Visuomotor impairment in early-stage Alzheimer’s disease: changes in relative timing of eye and hand movements. J Alzheimers Dis (2012) 30:131–43. 10.3233/JAD-2012-111883 22377783

[3] Caeyenberghs K van Roon D van Aken K De Cock P Linden CV Swinnen SP Static and dynamic visuomotor task performance in children with acquired brain injury: predictive control deficits under increased temporal pressure. J Head Trauma Rehabil (2009) 24:363–73. 10.1097/HTR.0b013e3181af0810 19858970

[4] Gao KL Ng SS Kwok JW Chow RT Tsang WW. Eye-hand coordination and its relationship with sensori-motor impairments in stroke survivors. J Rehabil Med (2010) 42:368–73. 10.2340/16501977-0520 20461340

[5] Procacci NM Stanford TR Wittenberg GF The relationship between visual orienting and interlimb synchrony in a patient with a superior parietal infarction: a case study. Neurocase (2009) 15:73–88. 10.1080/13554790802620558 19172431 PMC3557785

[6] Heitger MH Jones RD Anderson TJ. A new approach to predicting postconcussion syndrome after mild traumatic brain injury based upon eye movement function. Conf Proc IEEE Eng Med Biol Soc (2008) 2008:3570–3. 10.1109/IEMBS.2008.4649977 19163480

[7] Heitger MH Jones RD Macleod AD Snell DL Frampton CM Anderson TJ. Impaired eye movements in post-concussion syndrome indicate suboptimal brain function beyond the influence of depression, malingering or intellectual ability. Brain (2009) 132:2850–70. 10.1093/brain/awp181 19617197

[8] Dong W Yan B Johnson BP Millist L Davis S Fielding J Ischaemic stroke: the ocular motor system as a sensitive marker for motor and cognitive recovery. J Neurol Neurosurg Psychiatry (2013) 84:337–41. 10.1136/jnnp-2012-303926 23223333 PMC3582066

[9] O’Dell MW Lin CC Harrison V Stroke rehabilitation: strategies to enhance motor recovery. Annu Rev Med (2009) 60:55–68. 10.1146/annurev.med.60.042707.104248 18928333

[10] Beer RF Dewald JP Rymer WZ. Deficits in the coordination of multijoint arm movements in patients with hemiparesis: evidence for disturbed control of limb dynamics. Exp Brain Res (2000) 131:305–19. 10.1007/s002219900275 10789946

[11] Fisher BE Winstein CJ Velicki MR. Deficits in compensatory trajectory adjustments after unilateral sensorimotor stroke. Exp Brain Res (2000) 132:328–44. 10.1007/s002219900316 10883381

[12] McCrea PH Eng JJ. Consequences of increased neuromotor noise for reaching movements in persons with stroke. Exp Brain Res (2005) 162:70–7. 10.1007/s00221-004-2106-8 15536551 PMC3473069

[13] Tsang WW Ng SS Lee MW Tse SP Yip EW Yuen JK. Does postural stability affect the performance of eye-hand coordination in stroke survivors? Am J Phys Med Rehabil (2013) 92:781–8. 10.1097/PHM.0b013e3182876adb 23478460

[14] Velicki MR Winstein CJ Pohl PS. Impaired direction and extent specification of aimed arm movements in humans with stroke-related brain damage. Exp Brain Res (2000) 130:362–74. 10.1007/s002219900262 10706435

[15] Wenzelburger R Kopper F Frenzel A Stolze H Klebe S Brossmann A Hand coordination following capsular stroke. Brain (2005) 128:64–74. 10.1093/brain/awh317 15471902

[16] Zackowski KM Dromerick AW Sahrmann SA Thach WT Bastian AJ. How do strength, sensation, spasticity and joint individuation relate to the reaching deficits of people with chronic hemiparesis? Brain (2004) 127:1035–46. 10.1093/brain/awh116 14976070

[17] Leigh RJ Kennard C. Using saccades as a research tool in the clinical neurosciences. Brain (2004) 127:460–77. 10.1093/brain/awh035 14607787

[18] White OB Fielding J. Cognition and eye movements: assessment of cerebral dysfunction. J Neuroophthalmol (2012) 32:266–73. 10.1097/WNO.0b013e3182688230 22914692

[19] Anderson T Could saccadic function be a useful marker of stroke recovery? J Neurol Neurosurg Psychiatry (2013) 84:242 10.1136/jnnp-2012-304481 23334523

[20] Johansson RS Westling G Backstrom A Flanagan JR. Eye-hand coordination in object manipulation. J Neurosci (2001) 21:6917–32. 11517279 10.1523/JNEUROSCI.21-17-06917.2001 PMC6763066

[21] Flanagan JR Bowman MC Johansson RS. Control strategies in object manipulation tasks. Curr Opin Neurobiol (2006) 16:650–9. 10.1016/j.conb.2006.10.005 17084619

[22] Gaveau V Pisella L Priot AE Fukui T Rossetti Y Pelisson D Automatic online control of motor adjustments in reaching and grasping. Neuropsychologia (2014) 55:25–40. 10.1016/j.neuropsychologia.2013.12.005 24334110

[23] Prablanc C Echallier JE Jeannerod M Komilis E Optimal response of eye and hand motor systems in pointing at a visual target. II. Static and dynamic visual cues in the control of hand movement. Biol Cybern (1979) 35:183–7. 10.1007/BF00337063 518938

[24] Prablanc C Echallier JF Komilis E Jeannerod M. Optimal response of eye and hand motor systems in pointing at a visual target. I. Spatio-temporal characteristics of eye and hand movements and their relationships when varying the amount of visual information. Biol Cybern (1979) 35:113–24. 10.1007/BF00337063 518932

[25] Bogousslavsky J. Impairment of visually evoked eye movements with a unilateral parieto-occipital lesion. J Neurol (1987) 234:160–2. 10.1007/BF00314136 3585424

[26] Bogousslavsky J Meienberg O. Eye-movement disorders in brain-stem and cerebellar stroke. Arch Neurol (1987) 44:141–8. 10.1001/archneur.1987.00520140013011 3545158

[27] Catz A Ron S Solzi P Korczyn AD Saccade characteristics in patients with hemispheric stroke. J Neurol Rehabil (1997) 11:175–80.

[28] Colombo A Gibertoni M Sorgato P [Clinical study of disorders of eye movements (voluntary and following) after acute vascular accidents involving the cerebral hemispheres (author’s transl)]. Riv Patol Nerv Ment (1981) 102:38–46. 7345551

[29] Lekwuwa GU Barnes GR. Cerebral control of eye movements. II. Timing of anticipatory eye movements, predictive pursuit and phase errors in focal cerebral lesions. Brain (1996) 119(Pt 2):491–505. 10.1093/brain/119.2.491 8800944

[30] Pierrot-Deseilligny C Ploner CJ Muri RM Gaymard B Rivaud-Pechoux S. Effects of cortical lesions on saccadic eye movements in humans. Ann N Y Acad Sci (2002) 956:216–29. 10.1111/j.1749-6632.2002.tb02821.x 11960806

[31] Waespe W Baumgartner R. Enduring dysmetria and impaired gain adaptivity of saccadic eye movements in Wallenberg’s lateral medullary syndrome. Brain (1992) 115(Pt 4):1123–46. 10.1093/brain/115.4.1125 1393507

[32] Rivaud S Muri RM Gaymard B Vermersch AI Pierrot-Deseilligny C. Eye movement disorders after frontal eye field lesions in humans. Exp Brain Res (1994) 102:110–20. 10.1007/BF00232443 7895787

[33] Pierrot-Deseilligny C Rivaud S Gaymard B Agid Y. Cortical control of reflexive visually-guided saccades. Brain (1991) 114(Pt 3):1473–85. 10.1093/brain/114.3.1473 2065261

[34] Agrawal Y Schubert MC Migliaccio AA Zee DS Schneider E Lehnen N Evaluation of quantitative head impulse testing using search coils versus video-oculography in older individuals. Otol Neurotol (2014) 35:283–8. 10.1097/MAO.0b013e3182995227 24080977 PMC4532669

[35] Eggert T. Eye movement recordings: methods. Dev Ophthalmol (2007) 40:15–34. 10.1159/000100347 17314477

[36] Houben MM Goumans J van der Steen J. Recording three-dimensional eye movements: scleral search coils versus video oculography. Invest Ophthalmol Vis Sci (2006) 47:179–87. 10.1167/iovs.05-0234 16384960

[37] Imai T Sekine K Hattori K Takeda N Koizuka I Nakamae K Comparing the accuracy of video-oculography and the scleral search coil system in human eye movement analysis. Auris Nasus Larynx (2005) 32:3–9. 10.1016/j.anl.2004.11.009 15882818

[38] Kimmel DL Mammo D Newsome WT. Tracking the eye non-invasively: simultaneous comparison of the scleral search coil and optical tracking techniques in the macaque monkey. Front Behav Neurosci (2012) 6:49. 10.3389/fnbeh.2012.00049 22912608 PMC3418577

[39] McCamy MB Otero-Millan J Leigh RJ King SA Schneider RM Macknik SL Simultaneous recordings of human microsaccades and drifts with a contemporary video eye tracker and the search coil technique. PLoS One (2015) 10:e0128428. 10.1371/journal.pone.0128428 26035820 PMC4452707

[40] Stahl JS van Alphen AM De Zeeuw CI. A comparison of video and magnetic search coil recordings of mouse eye movements. J Neurosci Methods (2000) 99:101–10. 10.1016/S0165-0270(00)00218-1 10936649

[41] van der Geest JN Frens MA. Recording eye movements with video-oculography and scleral search coils: a direct comparison of two methods. J Neurosci Methods (2002) 114:185–95. 10.1016/S0165-0270(01)00527-1 11856570

[42] Yee RD Schiller VL Lim V Baloh FG Baloh RW Honrubia V. Velocities of vertical saccades with different eye movement recording methods. Invest Ophthalmol Vis Sci (1985) 26:938–44. 4008210

[43] Machado L Rafal RD. Control of fixation and saccades in humans with chronic lesions of oculomotor cortex. Neuropsychology (2004) 18:115–23. 10.1037/0894-4105.18.1.115 14744194

[44] Noudoost B Clark KL Moore T. A distinct contribution of the frontal eye field to the visual representation of saccadic targets. J Neurosci (2014) 34:3687–98. 10.1523/JNEUROSCI.3824-13.2014 24599467 PMC3942584

[45] Schall S Schmidt A [Dental assistants-continuing education in Munich (7)]. Quintessenz J (1991) 21:611–3. 1946966

[46] Louie K Grattan LE Glimcher PW. Reward value-based gain control: divisive normalization in parietal cortex. J Neurosci (2011) 31:10627–39. 10.1523/JNEUROSCI.1237-11.2011 21775606 PMC3285508

[47] Stuphorn V Brown JW Schall JD. Role of supplementary eye field in saccade initiation: executive, not direct, control. J Neurophysiol (2010) 103:801–16. 10.1152/jn.00221.2009 19939963 PMC2822692

[48] Stuphorn V Bauswein E Hoffmann KP. Neurons in the primate superior colliculus coding for arm movements in gaze-related coordinates. J Neurophysiol (2000) 83:1283–99. 10712456 10.1152/jn.2000.83.3.1283

[49] Hutton SB. Cognitive control of saccadic eye movements. Brain Cogn (2008) 68:327–40. 10.1016/j.bandc.2008.08.021 19028265

[50] Histed MH Pasupathy A Miller EK. Learning substrates in the primate prefrontal cortex and striatum: sustained activity related to successful actions. Neuron (2009) 63:244–53. 10.1016/j.neuron.2009.06.019 19640482 PMC2874751

[51] Pasupathy A Miller EK. Different time courses of learning-related activity in the prefrontal cortex and striatum. Nature (2005) 433:873–6. 10.1038/nature03287 15729344

[52] Thakkar KN van den Heiligenberg FM Kahn RS Neggers SF. Frontal-subcortical circuits involved in reactive control and monitoring of gaze. J Neurosci (2014) 34:8918–29. 10.1523/JNEUROSCI.0732-14.2014 24966390 PMC6608199

[53] Mahamed S Garrison TJ Shires J Basso MA. Stimulation of the substantia nigra influences the specification of memory-guided saccades. J Neurophysiol (2014) 111:804–16. 10.1152/jn.00002.2013 24259551 PMC3921393

[54] Watanabe M Munoz DP. Effects of caudate microstimulation on spontaneous and purposive saccades. J Neurophysiol (2013) 110:334–43. 10.1152/jn.00046.2013 23636720

[55] Gaymard B. Cortical and sub-cortical control of saccades and clinical application. Rev Neurol (Paris) (2012) 168:734–40. 10.1016/j.neurol.2012.07.016 22981301

[56] Shires J Joshi S Basso MA. Shedding new light on the role of the basal ganglia-superior colliculus pathway in eye movements. Curr Opin Neurobiol (2010) 20:717–25. 10.1016/j.conb.2010.08.008 20829033 PMC3008502

[57] Hikosaka O Takikawa Y Kawagoe R. Role of the basal ganglia in the control of purposive saccadic eye movements. Physiol Rev (2000) 80:953–78. 10893428 10.1152/physrev.2000.80.3.953

[58] Gaymard B Rivaud S Cassarini JF Dubard T Rancurel G Agid Y Effects of anterior cingulate cortex lesions on ocular saccades in humans. Exp Brain Res (1998) 120:173–83. 10.1007/s002210050391 9629959

[59] Belardinelli A Stepper MY Butz MV. It’s in the eyes: planning precise manual actions before execution. J Vis (2016) 16:18. 10.1167/16.1.18 26818971

[60] Hall J Guyton A Textbook of Medical Physiology. Philadelphia: W.B. Saunders (2005).

[61] Rathelot JA Strick PL. Muscle representation in the macaque motor cortex: an anatomical perspective. Proc Natl Acad Sci U S A (2006) 103:8257–62. 10.1073/pnas.0602933103 16702556 PMC1461407

[62] Bard C Fleury M Hay L Development of Eye Hand Coordination Across the Lifespan. Columbia, SC: University of South Carolina Press (1990).

[63] Crawford JD Medendorp WP Marotta JJ. Spatial transformations for eye-hand coordination. J Neurophysiol (2004) 92:10–9. 10.1152/jn.00117.2004 15212434

[64] Farber SD Assessing Neuromotor Performance Enablers. Thorofare, NJ: Slack (1991).

[65] Zoltan B Pedretti LW Evaluation of Muscle Tone and Coordination. St. Louis: C.V. Mosby (1990).

[66] Desrosiers J Hebert R Bravo G Dutil E. Upper-extremity motor co-ordination of healthy elderly people. Age Ageing (1995) 24:108–12. 10.1093/ageing/24.2.108 7793331

[67] Rizzo JR Hosseini M Wong EA Mackey WE Fung JK Ahdoot E The intersection between ocular and manual motor control: eye-hand coordination in acquired brain injury. Front Neurol (2017) 8:227 10.3389/fneur.2017.00227 28620341 PMC5451505

[68] Edlin JM Leppanen ML Fain RJ Hacklander RP Hanaver-Torrez SD Lyle KB. On the use (and misuse?) of the Edinburgh Handedness Inventory. Brain Cogn (2015) 94:44–51. 10.1016/j.bandc.2015.01.003 25656540

[69] Carlson RV Boyd KM Webb DJ. The revision of the declaration of Helsinki: past, present and future. Br J Clin Pharmacol (2004) 57:695–713. 10.1111/j.1365-2125.2004.02103.x 15151515 PMC1884510

[70] Eckenwiler LA Ells C Feinholz D Schonfeld T Hopes for Helsinki: reconsidering “vulnerability”. J Med Ethics (2008) 34:765–6. 10.1136/jme.2007.023481 18827112

[71] Schmidt H Mehring S McMillan J Interpreting the declaration of Helsinki (2008): “must”, “should” and different kinds of obligation. Med Law (2010) 29:565–91. 22145551

[72] Fugl-Meyer AR Jaasko L Leyman I Olsson S Steglind S. The post-stroke hemiplegic patient. 1. A method for evaluation of physical performance. Scand J Rehabil Med (1975) 7:13–31. 1135616

[73] Srivastava A Rapoport MJ Leach L Phillips A Shammi P Feinstein A. The utility of the mini-mental status exam in older adults with traumatic brain injury. Brain Inj (2006) 20:1377–82. 10.1080/02699050601111385 17378229

[74] Rankin J Cerebral vascular accidents in patients over the age of 60. I. General considerations. Scott Med J (1957) 2:127–36. 10.1177/003693305700200401 13432825

[75] Volz M Mobus J Letsch C Werheid K. The influence of early depressive symptoms, social support and decreasing self-efficacy on depression 6 months post-stroke. J Affect Disord (2016) 206:252–5. 10.1016/j.jad.2016.07.041 27513631

[76] Zagar R Mead JD. Analysis of a short test battery for children. J Clin Psychol (1983) 39:590–7. 10.1002/1097-4679(198307)39:4<590::AID-JCLP2270390422>3.0.CO;2-B 6875000

[77] Malloy P Belanger H Hall S Aloia M Salloway S. Assessing visuoconstructional performance in AD, MCI and normal elderly using the Beery Visual-Motor Integration Test. Clin Neuropsychol (2003) 17:544–50. 10.1076/clin.17.4.544.27947 15168918

[78] Temple V Drummond C Valiquette S Jozsvai E. A comparison of intellectual assessments over video conferencing and in-person for individuals with ID: preliminary data. J Intellect Disabil Res (2010) 54:573–7. 10.1111/j.1365-2788.2010.01282.x 20576065

[79] Tannenbaum S The eye chart and Dr. Snellen. J Am Optom Assoc (1971) 42:89–90. 4925668

[80] Beck RW Bergstrom TJ Lichter PR. A clinical comparison of visual field testing with a new automated perimeter, the Humphrey Field Analyzer, and the Goldmann perimeter. Ophthalmology (1985) 92:77–82. 10.1016/S0161-6420(85)34065-4 3974997

[81] Schenkenberg T Bradford DC Ajax ET. Line bisection and unilateral visual neglect in patients with neurologic impairment. Neurology (1980) 30:509–17. 10.1212/WNL.30.5.509 7189256

[82] Johnston CW Diller L. Exploratory eye movements and visual hemi-neglect. J Clin Exp Neuropsychol (1986) 8:93–101. 10.1080/01688638608401299 3944247

[83] Mangione CM Lee PP Gutierrez PR Spritzer K Berry S Hays RD Development of the 25-item National Eye Institute Visual Function Questionnaire. Arch Ophthalmol (2001) 119:1050–8. 10.1001/archopht.119.7.1050 11448327

[84] van Boxtel JJ Lu H A biological motion toolbox for reading, displaying, and manipulating motion capture data in research settings. J Vis (2013) 13:7 10.1167/13.12.7 24130256

[85] LeVasseur AL Flanagan JR Riopelle RJ Munoz DP. Control of volitional and reflexive saccades in Tourette’s syndrome. Brain (2001) 124:2045–58. 10.1093/brain/124.10.2045 11571221

[86] Rizzo J-R Hudson TE Abdou A Lui YW Rucker JC Raghavan P Disrupted saccade control in chronic cerebral injury: upper motor neuron-like disinhibition in the ocular motor system. Front Neurol (2017) 8:12 10.3389/fneur.2017.00012 28184211 PMC5266728

[87] Carlton LG Visual information: the control of aiming movements. Q J Exp Psychol (1981) 33:87–93. 10.1080/14640748108400771

[88] Binsted G Elliott D. Ocular perturbations and retinal/extraretinal information: the coordination of saccadic and manual movements. Exp Brain Res (1999) 127:193–206. 10.1007/s002210050789 10442411

[89] Helsen WF Elliott D Starkes JL Ricker KL. Temporal and spatial coupling of point of gaze and hand movements in aiming. J Mot Behav (1998) 30:249–59. 10.1080/00222899809601340 20037082

[90] Helsen WF Elliott D Starkes JL Ricker KL. Coupling of eye, finger, elbow, and shoulder movements during manual aiming. J Mot Behav (2000) 32:241–8. 10.1080/00222890009601375 10975272

[91] Starkes J Helsen W Elliott D A menage a trois: the eye, the hand and on-line processing. J Sports Sci (2002) 20:217–24. 10.1080/026404102317284772 11999477

[92] Hayhoe MM Shrivastava A Mruczek R Pelz JB. Visual memory and motor planning in a natural task. J Vis (2003) 3:49–63. 10.1167/3.1.6 12678625

[93] Irwin DE. Information integration across saccadic eye movements. Cogn Psychol (1991) 23:420–56. 10.1016/0010-0285(91)90015-G 1884598

[94] Ballard DH Hayhoe MM Pelz JB. Memory representations in natural tasks. J Cogn Neurosci (1995) 7:66–80. 10.1162/jocn.1995.7.1.66 23961754

[95] Irwin DE Integrating information across saccadic eye movements. Curr Dir Psychol Sci (1996) 5:94–100. 10.1111/1467-8721.ep10772833

[96] O’Regan JK Solving the “real” mysteries of visual perception: the world as an outside memory. Can J Psychol (1992) 46:461–88. 10.1037/h0084327 1486554

[97] Land M Mennie N Rusted J. The roles of vision and eye movements in the control of activities of daily living. Perception (1999) 28:1311–28. 10.1068/p2935 10755142

[98] Ballard DH Animate vision. Artif Intell (1991) 48:57–86. 10.1016/0004-3702(91)90080-4

[99] Cilo M Politzer T Ripley DL Weintraub A Vision examination of TBI patients in an acute rehabilitation hospital. NeuroRehabilitation (2010) 27:237–42. 10.3233/NRE-2010-0603 21098992

[100] Brouwer AM Franz VH Gegenfurtner KR. Differences in fixations between grasping and viewing objects. J Vis (2009) 9:18.1–24. 10.1167/9.1.18 19271888

[101] Cavina-Pratesi C Hesse C Why do the eyes prefer the index finger? Simultaneous recording of eye and hand movements during precision grasping. J Vis (2013) 13:15 10.1167/13.5.15 23599419

[102] van Donkelaar P Siu KC Walterschied J. Saccadic output is influenced by limb kinetics during eye-hand coordination. J Mot Behav (2004) 36:245–52. 10.3200/JMBR.36.3.245-252 15262621

[103] Ohtsuka K Noda H. The effect of microstimulation of the oculomotor vermis on discharges of fastigial neurons and visually-directed saccades in macaques. Neurosci Res (1991) 10(4):290–5. 10.1016/0168-0102(91)90086-E 1652724

[104] Keating EF Kenney DV Gooley SG Pratt SE McGillis SL. Targeting errors and reduced oculomotor range following ablations of the superior colliculus or pretectum/thalamus. Behav Brain Res (1986) 22:191–210. 10.1016/0166-4328(86)90064-1 3790242

[105] Pierrot-Deseilligny C Rosa A Masmoudi K Rivaud S Gaymard B. Saccade deficits after a unilateral lesion affecting the superior colliculus. J Neurol Neurosurg Psychiatry (1991) 54(12):1106–9. 10.1136/jnnp.54.12.1106 1783927 PMC1014690

[106] Hudson TE Maloney LT Landy MS. Movement planning with probabilistic target information. J Neurophysiol (2007) 98:3034–46. 10.1152/jn.00858.2007 17898140 PMC2638584

[107] Mann DT Williams AM Ward P Janelle CM. Perceptual-cognitive expertise in sport: a meta-analysis. J Sport Exerc Psychol (2007) 29:457–78. 10.1123/jsep.29.4.457 17968048

[108] McRobert AP Williams AM Ward P Eccles DW. Tracing the process of expertise in a simulated anticipation task. Ergonomics (2009) 52:474–83. 10.1080/00140130802707824 19401899

[109] Piras A Lobietti R Squatrito S. A study of saccadic eye movement dynamics in volleyball: comparison between athletes and non-athletes. J Sports Med Phys Fitness (2010) 50:99–108. 20308980

[110] Willams AM Hodges NJ North JS Barton G. Perceiving patterns of play in dynamic sport tasks: investigating the essential information underlying skilled performance. Perception (2006) 35:317–32. 10.1068/p5310 16619949

[111] Williams AM. Perceptual skill in soccer: implications for talent identification and development. J Sports Sci (2000) 18:737–50. 10.1080/02640410050120041 11043899

[112] Savelsbergh GJ Williams AM Van der Kamp J Ward P. Visual search, anticipation and expertise in soccer goalkeepers. J Sports Sci (2002) 20:279–87. 10.1080/026404102317284826 11999482

[113] Savelsbergh GJ Van der Kamp J Williams AM Ward P. Anticipation and visual search behaviour in expert soccer goalkeepers. Ergonomics (2005) 48:1686–97. 10.1080/00140130500101346 16338733

[114] Battaglia-Mayer A Archambault PS Caminiti R. The cortical network for eye-hand coordination and its relevance to understanding motor disorders of parietal patients. Neuropsychologia (2006) 44:2607–20. 10.1016/j.neuropsychologia.2005.11.021 16458334

[115] Caeyenberghs K Leemans A Geurts M Taymans T Vander Linden C Smits-Engelsman BC Brain-behavior relationships in young traumatic brain injury patients: fractional anisotropy measures are highly correlated with dynamic visuomotor tracking performance. Neuropsychologia (2010) 48:1472–82. 10.1016/j.neuropsychologia.2010.01.017 20117121

[116] Hudson TE Maloney LT Landy MS. Optimal compensation for temporal uncertainty in movement planning. PLoS Comput Biol (2008) 4:e1000130. 10.1371/journal.pcbi.1000130 18654619 PMC2442880

[117] Cirstea MC Levin MF Compensatory strategies for reaching in stroke. Brain (2000) 123(Pt 5):940–53. 10.1093/brain/123.5.940 10775539

[118] Ketcham CJ Rodriguez TM Zihlman KA. Targeted aiming movements are compromised in nonaffected limb of persons with stroke. Neurorehabil Neural Repair (2007) 21:388–97. 10.1177/1545968306297872 17369510

[119] Kusoffsky A Apel I Hirschfeld H. Reaching-lifting-placing task during standing after stroke: coordination among ground forces, ankle muscle activity, and hand movement. Arch Phys Med Rehabil (2001) 82:650–60. 10.1053/apmr.2001.22611 11346843

[120] Takahashi CD Reinkensmeyer DJ. Hemiparetic stroke impairs anticipatory control of arm movement. Exp Brain Res (2003) 149:131–40. 10.1007/s00221-002-1340-1 12610680

[121] Beer R Dewald J Rymer Z Disturbances of voluntary movement coordination in stroke: problems of planning or execution? Prog Brain Res (1999) 123:455–60. 10.1016/S0079-6123(08)62881-2 10635741

[122] Rizzo JR Hudson TE Abdou A Rashbaum IG George AE Raghavan P Motor planning poststroke: impairment in vector-coded reach plans. Physiol Rep (2015) 3:e12650 10.14814/phy2.12650 26660558 PMC4760446

[123] Raghavan P Krakauer JW Gordon AM. Impaired anticipatory control of fingertip forces in patients with a pure motor or sensorimotor lacunar syndrome. Brain (2006) 129:1415–25. 10.1093/brain/awl070 16597653 PMC2093998

[124] Raghavan P Santello M Gordon AM Krakauer JW. Compensatory motor control after stroke: an alternative joint strategy for object-dependent shaping of hand posture. J Neurophysiol (2010) 103:3034–43. 10.1152/jn.00936.2009 20457866 PMC2888236

[125] Rizzo JR Hudson TE Dai W Birkemeier J Pasculli RM Selesnick I Rapid number naming in chronic concussion: eye movements in the King-Devick test. Ann Clin Transl Neurol (2016) 3(10):801–11. 10.1002/acn3.345 27752515 PMC5048390

[126] Rizzo JR Hudson TE Dai W Desai N Yousefi A Palsana D Objectifying eye movements during rapid number naming: methodology for assessment of normative data for the King-Devick test. J Neurol Sci (2016) 362:232–9. 10.1016/j.jns.2016.01.045 26944155 PMC4821571

[127] Contreras R Ghajar J Bahar S Suh M. Effect of cognitive load on eye-target synchronization during smooth pursuit eye movement. Brain Res (2011) 1398:55–63. 10.1016/j.brainres.2011.05.004 21620377

[128] Maruta J Suh M Niogi SN Mukherjee P Ghajar J. Visual tracking synchronization as a metric for concussion screening. J Head Trauma Rehabil (2010) 25:293–305. 10.1097/HTR.0b013e3181e67936 20611047

[129] Suh M Basu S Kolster R Sarkar R McCandliss B Ghajar J Increased oculomotor deficits during target blanking as an indicator of mild traumatic brain injury. Neurosci Lett (2006) 410:203–7. 10.1016/j.neulet.2006.10.001 17055156

[130] Suh M Kolster R Sarkar R McCandliss B Ghajar J Cognitive and Neurobiological Research Consortium. Deficits in predictive smooth pursuit after mild traumatic brain injury. Neurosci Lett (2006) 401:108–13. 10.1016/j.neulet.2006.02.074 16554121

[131] Heitger MH Anderson TJ Jones RD Dalrymple-Alford JC Frampton CM Ardagh MW. Eye movement and visuomotor arm movement deficits following mild closed head injury. Brain (2004) 127:575–90. 10.1093/brain/awh066 14736751

[132] Heitger MH Jones RD Dalrymple-Alford JC Frampton CM Ardagh MW Anderson TJ Mild head injury – a close relationship between motor function at 1 week post-injury and overall recovery at 3 and 6 months. J Neurol Sci (2007) 253:34–47. 10.1016/j.jns.2006.11.007 17207818

[133] Heitger MH Jones RD Dalrymple-Alford JC Frampton CM Ardagh MW Anderson TJ. Motor deficits and recovery during the first year following mild closed head injury. Brain Inj (2006) 20:807–24. 10.1080/02699050600676354 17060148

[134] Galetta KM Chapman KR Essis MD Alosco ML Gillard D Steinberg E Screening utility of the King-Devick test in mild cognitive impairment and Alzheimer disease dementia. Alzheimer Dis Assoc Disord (2016) 31(2):152–8. 10.1097/WAD.0000000000000157 PMC5154783 27299935

[135] Boxer AL Garbutt S Seeley WW Jafari A Heuer HW Mirsky J Saccade abnormalities in autopsy-confirmed frontotemporal lobar degeneration and Alzheimer disease. Arch Neurol (2012) 69:509–17. 10.1001/archneurol.2011.1021 22491196 PMC3423186

[136] Garbutt S Matlin A Hellmuth J Schenk AK Johnson JK Rosen H Oculomotor function in frontotemporal lobar degeneration, related disorders and Alzheimer’s disease. Brain (2008) 131:1268–81. 10.1093/brain/awn047 18362099 PMC2367697

[137] Heuer HW Mirsky JB Kong EL Dickerson BC Miller BL Kramer JH Antisaccade task reflects cortical involvement in mild cognitive impairment. Neurology (2013) 81:1235–43. 10.1212/WNL.0b013e3182a6cbfe 23986300 PMC3795604

[138] Lee D Poizner H Corcos DM Henriques DY. Unconstrained reaching modulates eye-hand coupling. Exp Brain Res (2014) 232:211–23. 10.1007/s00221-013-3732-9 24121521 PMC3945424

[139] Gorbet DJ Sergio LE. The behavioural consequences of dissociating the spatial directions of eye and arm movements. Brain Res (2009) 1284:77–88. 10.1016/j.brainres.2009.05.057 19497310

[140] Kaplan J Hier DB. Visuospatial deficits after right hemisphere stroke. Am J Occup Ther (1982) 36:314–21. 10.5014/ajot.36.5.314 7091293

[141] Coutte A Olivier G Faure S Baccino T. Preparation of forefinger’s sequence on keyboard orients ocular fixations on computer screen. Cogn Process (2014) 15:415–22. 10.1007/s10339-014-0612-6 24682551

[142] Rand MK Stelmach GE. Effects of hand termination and accuracy requirements on eye-hand coordination in older adults. Behav Brain Res (2011) 219:39–46. 10.1016/j.bbr.2010.12.008 21163306 PMC3062752

[143] Khan A Song J McPeek R. The eye dominates in guiding attention during simultaneous eye and hand movements. J Vis (2011) 11:9. 10.1167/11.1.9 21216769 PMC3141209

[144] Song JH McPeek RM. Eye-hand coordination during target selection in a pop-out visual search. J Neurophysiol (2009) 102:2681–92. 10.1152/jn.91352.2008 19726722 PMC2777825

